# A Method for Combining Thin and Thick Malleable Titanium Mesh in the Repair of Cranial Defects

**DOI:** 10.7759/cureus.267

**Published:** 2015-05-05

**Authors:** Darryl Lau, Michael W McDermott

**Affiliations:** 1 Department of Neurological Surgery, University of California, San Francisco; 2 Department of Neurological Surgery, Carol Franc Buck Breast Care, University of California, San Francisco

**Keywords:** cranial defect, titanium mesh, meningioma, pterion, cranioplasty, reconstruction

## Abstract

Introduction: Cranial defects following the removal of tumor involved bone require repair and reconstruction for brain protection and cosmesis. A variety of autologous bone substrates and synthetic materials can be employed, alone or in combination. In this article, we describe the use of dual thin and thick titanium mesh, connected together using plate hardware, to repair a right frontotemporal sphenoidal bone defect following resection of a hyperostosing sphenoid wing meningioma.

Methods: Reconstruction of the pterion was done with a dual mesh cranioplasty. After replacement of the native orbitozygomatic and frontotemporal bone pieces, a piece of thinner mesh was molded to the pterional defect connecting the two bone pieces and re-creating the concave shape of the pterion below the superior temporal line. The circular area of the bony defect overlying the frontal and temporal lobes was supplemented by cutting and molding an additional piece of thicker mesh which was secured to the thinner mesh with burr hole cover sectors using rescue screws.

Results: A 30-year-old woman presented with painless proptosis and was found to have a hyperostosing right sphenoid wing meningioma. The patient underwent a frontotemporal orbitozygomatic craniotomy for tumor resection and extensive bony osteotomy. Repair and reconstruction of the cranial defect in the region were accomplished at the time of open operation using two thicknesses of mesh connected one to another with titanium plate pieces and rescue screws. The patient underwent gross total resection of the meningioma and near total resection of the soft tissue and bony components (Simpson Grade II). The external cosmetic results following the orbital-cranial reconstruction with the dual mesh technique was deemed “very good” by the surgeon and patient. Postoperative CT imaging demonstrated symmetric re-approximation of the shape of the pterion as compared to the opposite side.

Conclusions: We present a method for connecting two titanium mesh sheets with available hardware to improve the strength in compression while maintaining the ability to mold thinner sheets as necessary for the best cosmetic results. This method is an option for coverage of bony defects in the region of the pterion for young, physically active patients providing them with additional mesh cranioplasty strength.

## Introduction

The removal of tumor-involved bone in surgery for hyperostosing meningiomas of the sphenoid wing is necessary to improve the postoperative cosmetic result and to reduce the chance of tumor recurrence [1-3]. At times, hyperostosis associated with meningiomas can be extensive and involve a large portion of the skull; thus, after resection and osteotomy, defects can be large. One of the most challenging areas to reconstruct is the orbit and fronto-temporal-sphenoidal region, given the bony shapes in the region are both concave and convex in close proximity [4]. It can be technically difficult to recreate these shapes after craniectomy to ensure a good cosmetic result while providing adequate strength for brain protection.

A variety of options exist for the surgeon to repair cranial defects of the pterion using both autologous and synthetic materials [5-9]. In this technical report, we describe a two-part mesh cranioplasty using a thin and thick mesh, connected one to another with existing hardware, to reconstruct the pterion. The first thinner mesh is molding over the curvilinear shapes of the pterion, connecting adjacent bone pieces for more rigid fixation. A template of the underlying skull defect is made and used to cut a thicker mesh, which can be secured with sector plate pieces and rescue screws to the molded thinner mesh.

## Technical report

### Double mesh cranioplasty: Surgical technique

**Figure 1:**
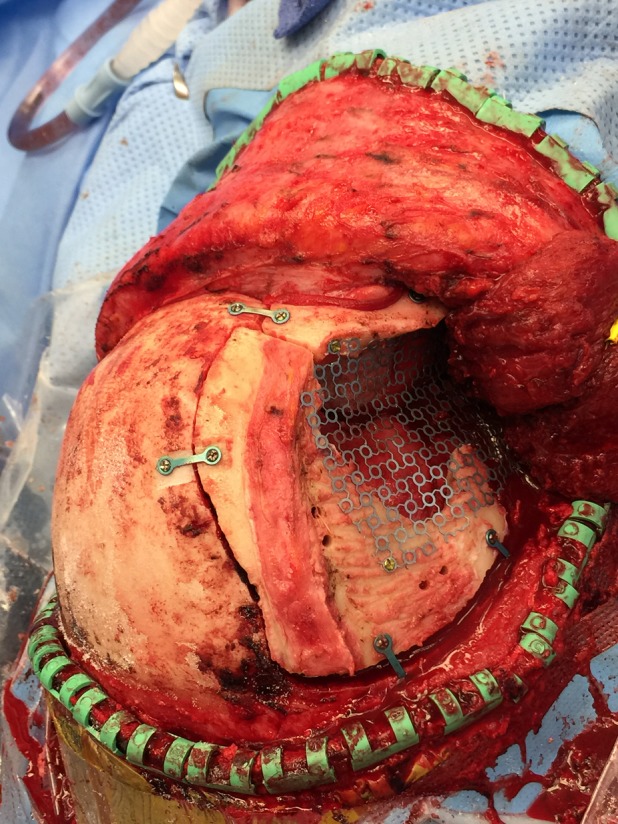
Thinner titanium mesh molded to replicate the concavity of the temporalis fossa

Following replacement of the orbital zygomatic osteotomy bone piece and the frontal temporal bone flap, a piece of thinner titanium mesh was molded by hand to replicate the concavity of the temporalis fossa while securing the two bone pieces together as well as to the bone near the floor of the middle fossa (Figure 1).

**Figure 2:**
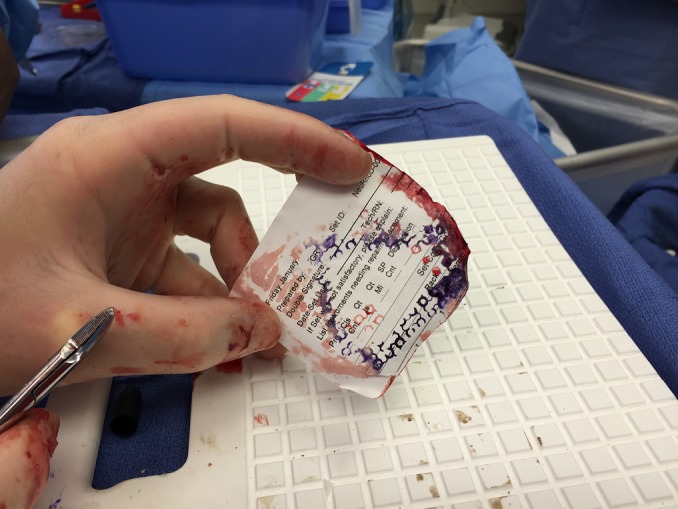
Outline of the bony defect for guide of thick mesh creation

This molded mesh was then removed and the bone edges were marked with a surgical marker so that a template of the defect could be created by placing a piece paper over top and using this to outline the thicker piece of gold mesh to the appropriate size to cover just the bone defect (Figure 2).

**Figure 3:**
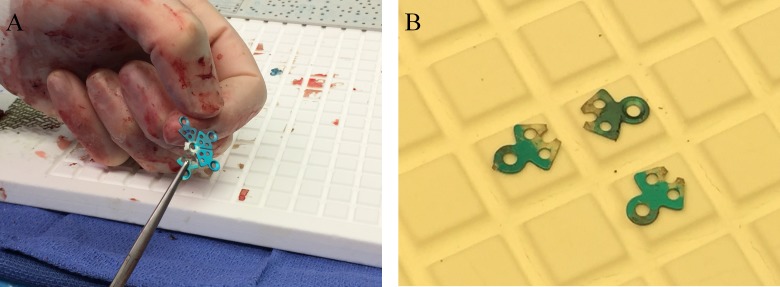
Obtaining sectors form burr-hole covers

The thicker mesh was then contoured by hand to try and match the shape of the thinner blue mesh. Next, a small burr hole cover was used as a donor for three small sectors; the sectors were broken off using a hinge motion until the metal fatigued at the bending point (Figures 3A, 3B).

**Figure 4:**
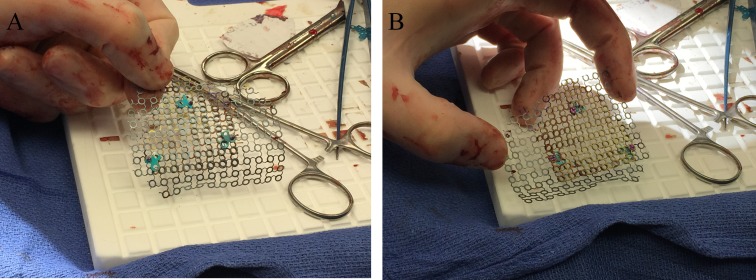
Securing thin and thick mesh with sector plates

The sector plates were placed behind the thinner mesh, and the plate holes (not the screw holes) were used to receive the larger rescue screws (Figures 4A, 4B).

**Figure 5:**
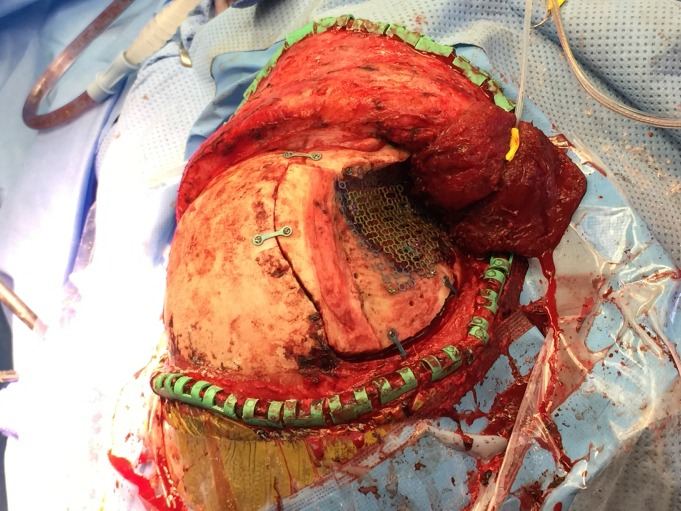
Re-implantation of dual mesh into defect site

The composite mesh cranioplasty was then re-secured to the surrounding skull using the initial thin mesh screw holes (Figure 5).

### Outcome

**Figure 6:**
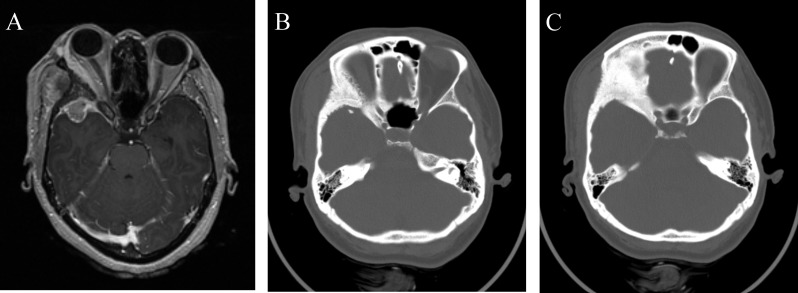
Preoperative MRI and CT imaging of right middle fossa meningioma (A) Axial T1-weight MRI with contrast revealing a right middle fossa meningioma with associated hyperostosis of surrounding bone and invasion of the temporalis muscle. (B and C) Extensive bony hyperostosis of the right lateral orbit and pterion.

A 30-year-old female presented with painless right eye proptosis and was found to have a right middle fossa meningioma with extensive hyperostosis of surrounding bone with extension into the temporalis muscle (Figure 6). Informed patient consent was obtained for this study.

**Figure 7:**
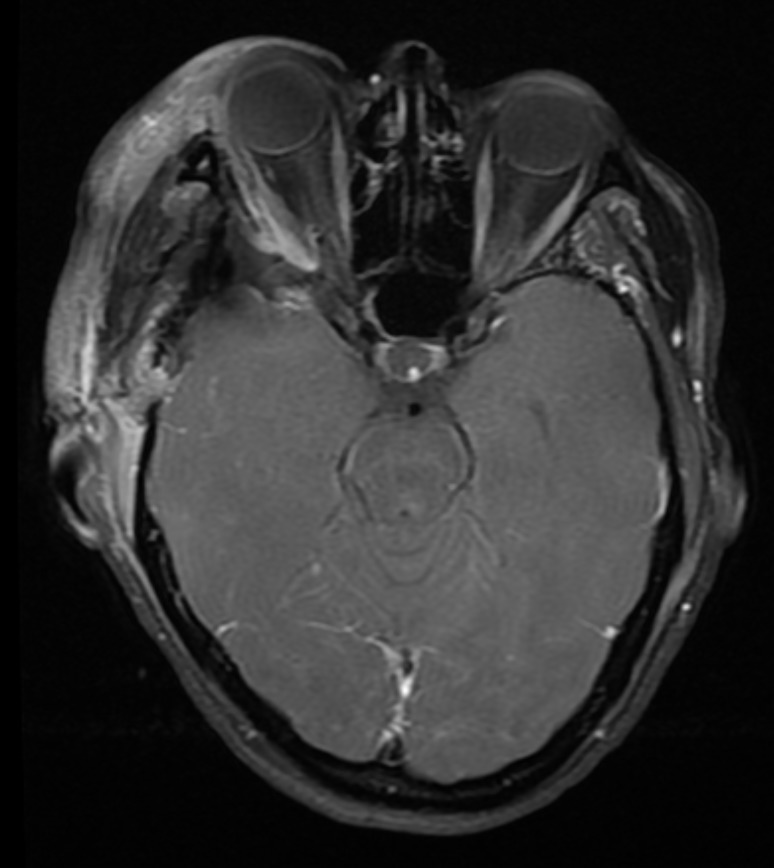
Postoperative MRI following right orbitozygomatic craniotomy for tumor resection Axial T1-weighted MRI with contrast showing near total resection.

**Figure 8:**
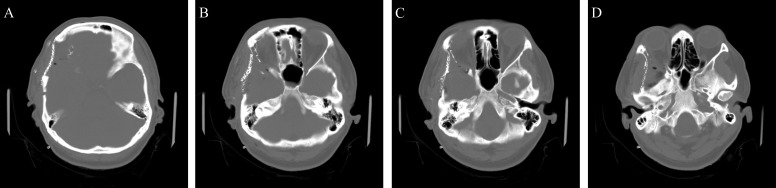
Postoperative CT following right orbitozygomatic craniotomy and cranial reconstruction

The patient was taken to the operating room for a right frontotemporal orbitozygomatic craniotomy for tumor resection. Pathology revealed a meningioma without high-grade features consistent with World Health Organization (WHO) Grade II. Given extensive hyperostosis and invasion of the temporalis muscle, osteotomy of surrounding bone left the patient with a large defect in the area of the orbit and pterion. We utilized the dual mesh technique to reconstruct the curvature of the pterion. Surgical removal of the soft tissue tumor and bone were near total (Figure 7). A postoperative CT scan demonstrated symmetric re-approximation of the shape of the pterion as compared to the opposite side on postoperative CT imaging (Figure 8). At follow-up, the external cosmetic results of the surgery were deemed “very good” by the surgeon and patient.

## Discussion

The challenges of meningioma surgery are often related to the location, vascularity, tumor consistency, and the presence of bony hyperostosis or invasion of surrounding tissue. It is well known that obtaining a more complete resection, according to the Simpson Grading System, is associated with lower rates of recurrence [3]. Therefore, in the setting of extensive bony and soft tissue involvement needing aggressive resection, patients are left with cranial defects requiring reconstruction. Proper reconstruction of the area around the orbit and pterion can be particularly difficult given the concave and convex nature of the area. The main objectives of cranioplasty are to promote primary wound healing, obliterate dead space, restore the normal barriers protecting the intracranial structures, and obtain a satisfactory cosmetic result.

Repairing and reconstructing a cranial defect during a patient’s first operation for a tumor involving bone can be done with autologous bone or synthetic implants. Autologous bone can be harvested from adjacent calvarium (preferably from areas with similar contours) and implanted into the cranial defects [10-12]. The outcomes are good when performed by experienced surgeons. However, this technique is formidable and can be technically difficult. Harvest of the autologous bone requires additional bone removal, splitting of bone, shaping, and fixation of the harvested bone pieces to replicate the bone removed from the tumor site. The additional craniotomy site where the bone is harvested may increase the risk for complications, such as an epidural hematoma formation [13].

Synthetic materials can be used alone or in combination. Authors Bloch and McDermott described an in-situ cranioplasty where the abnormal bone could be shaped to a more normal contour and then used to mold mesh to the proper curvature before combining it with methyl methacrylate [14]. Methyl methacrylate is strong in compression but weak in tension while the reverse is true for titanium mesh. Combining the two together yields additional strength with properties of both elements, similar to the effects of adding rebar to concrete. Titanium mesh can be used alone and is supplied in varying thicknesses and sizes. Thicker mesh is difficult to mold to a multi-curved surface, and often the postoperative appearance on CT scans appears “flat” as compared to the intraoperative impression of the adequacy of shape.

Porous polyethylene implants are also available, with or without impregnated mesh [7, 15]. These implants may be weaker in compression than other single compound materials but are designed to promote tissue ingrowth and may reduce seroma formation [16]. Custom molded cranioplasty implants are made of metal or synthetic polymers but require a pre-existing cranial defect for modeling. This would require a two-stage procedure; the first to excise tumor-involved bone with subsequent 3D imaging, computer-assisted design modeling, and creation of the cranioplasty. Next, a second stage procedure is needed for implantation of the custom implant.

In the dual mesh cranioplasty technique, thin mesh is used first to contour the desired shape more easily while the thicker mesh is used only over the bone defect site covering a smaller area without attempts to shape it to cover or recreate irregular surfaces. Connecting the two pieces together cannot be done without using some additional hardware, and to our knowledge, no plate manufacturer makes such an add-on component for this purpose. The senior author discovered by chance that the holes in the sectors of burr hole covers will accommodate a wider rescue screw in the same set, thus providing the “nut” (sector plate form burr hole cover) and “bolt” (rescue screw) for fixing the plates together. The resulting mesh cranioplasty is stronger in compression over the bony defect site and may provide additional protection for young, physically active patients who require craniectomy as part of their initial treatment.

## Conclusions

The composite dual mesh cranioplasty allows the maintenance of a cosmetically appropriate skull surface shape for the temporalis fossa, and connect the orbital osteotomy bone piece to the pterional bone flap, while adding strength in compression for the area of the skull defect over the frontal and temporal lobes. Importantly, it can be done during the first surgery, utilizing regularly available implants, and offers good cosmesis for the patient.
